# The Influence of Partial Knee Replacement Designs on Tensile Strain at Implant-Bone Interface

**DOI:** 10.1155/2012/607872

**Published:** 2012-05-17

**Authors:** He Wang, Lindsey Rolston

**Affiliations:** ^1^Biomechanics Laboratory, School of Physical Education, Sport, and Exercise Science, Ball State University, Muncie, IN 47306, USA; ^2^Henry County Center for Orthopedics Surgery & Sports Medicine, New Castle, IN 47362, USA

## Abstract

Partial knee replacement (PKR) results in fast recovery and good knee mechanics and is ideal to treat medial knee osteoarthritis. Cementless PKR depends on bone growing into the implant surface for long-term fixation. Implant loosening may occur due to high tensile strain resulted from large mechanical loads during rehab exercises. The purpose of this study is to investigate whether external fixations such as superior screw and frontal flange could reduce the tensile strain at the implant-bone interface. Three medial PKRs were designed. The first PKR had no external fixations. A superior screw and a frontal flange were then added to the first PKR to form the second and third PKR designs, respectively. Finite element analysis was performed to examine the tensile strain at the implant-bone interface during weight-bearing exercises. The PKR with no external fixations exhibited high tensile strain at the anterior implant-bone interface. Both the screwed and flanged PKRs effectively reduced the tensile strain at the anterior implant-bone interface. Furthermore, the flanged PKR resulted in a more uniform reduction of the tensile strain than the screwed PKR. In conclusion, external fixations are necessary to alleviate tensile strain at the implant-bone interface during knee rehab exercises.

## 1. Introduction

Osteoarthritis (OA) is a cartilage degenerative disease and causes more disability with respect to mobility than any other single disease in the elderly [1]. Knee OA typically affects joints in a nonuniform manner; the medial compartment of the knee is most frequently affected in both men and women [2]. Furthermore, the three most common areas of knee OA distribution are medial compartment, patellofemoral compartment, and medial/patellofemoral compartment overlap [3]. The predominance of medial knee OA is likely due to the high medial forces generated during weight-bearing activities (e.g., walking) [4, 5]. It is believed that the increased knee varus loading is strongly associated with risk of medial knee OA progression [6]. In fact, a radiographic study shows that varus knee alignment (bowlegged) increases medial knee OA progression in as little as 18 months [7].

Medial knee OA causes severe knee pain and knee stiffness, reduces knee function, and leads to disability [1, 8]. The most common surgical treatment of medial knee OA is total knee replacement (TKR) [9]. TKR has been the gold standard for treatment of knee OA because it results in excellent pain relief and has a long-term success rate (10–15 years) [10, 11]. However, TKR alternates the entire knee articular geometry and sacrifices the cruciate ligaments, which often leads to abnormal knee kinematics [12, 13]. It is ideal to only replace the affected knee compartments and reserve other intact portions and cruciate ligaments because the damage associated with medial knee OA is often limited to cartilage degeneration in the medial and patellofemoral compartments [3]. One alternative to the TKR, especially for more active patients, is partial knee replacement (PKR), which leaves the lateral knee compartment and cruciate ligaments intact while replacing the affected knee compartments (e.g., medial compartment and patellofemoral joint). PKR results in fast recovery times, less bone loss and normal knee mechanics [8, 12–15].

Adequate fixation of PKR tibial tray in the bone is important for reproducing normal knee kinematics. There are two major fixation techniques available for PKR. For three decades, fast-curing bone cement has been used to glue the knee implant to the bone for both TKR and PKR designs. Cement provides quick fixation of the tibial tray to the bone. However, the contact area between the PKR implant and bone is less than half of that of the TKR. Due to the small contact area between the PKR implant and bone, it is not uncommon to observe implant loosening and migration after one or two years of usage. Cementless fixation becomes popular in recent years. Cementless fixation features in promoting bone growth into the implant-bone interface for stability. The implant-bone contact areas include bottom surface of the tibial tray and surface of the press-fit pegs. These surfaces are covered with porous coating of sintered beads. New bone tissue can easily grow into the coating and form a reliable long-term fixation. Pandit et al. reported that the bone in-growth at the implant-bone interface is satisfactory at one year of postsurgery [16].

When cementless fixation is in use, it normally takes two to three months for the bone to remodel and replace the damaged tissue at the implant-bone interface and fully grow into the implant surface. During this process, bone strength is weakened; bone tissue lacks the ability of stabilizing the tibial tray. Thus, mechanical loading from knee rehab exercises may result in early loosening of the tibia tray. Migration of the tibial tray due to loosening may further lead to abnormal knee kinematics and eventually failure of the knee replacement. To date, the effect of mechanical loads from knee exercises on bone stress/strain at implant-bone interface has yet to be determined. Quantifying bone strain at implant-bone interface is essential for designing better PKRs to resist implant loosening.

Normal knee flexion is associated with femoral rollback with regard to the tibia [17]. During exercises involved knee flexion such as sit-to-stand, stair climbing, squat, and lunge, the femorotibial contact point is in the posterior region of the tibial plateau [18, 19]. Thus, axial mechanical loads during knee exercises are applied in the posterior region of the tibial plateau. As normal knee kinematics aree associated with PKR knees, the mechanical load from knee exercises is expected to be applied in the posterior region of the tibial tray. Therefore, it is possible that the tibia could experience a large bending load and the anterior implant-bone interface could be subjected to high tensile forces, which may result in early implant loosening. Therefore, introducing external fixation mechanisms to the PKR may reduce the risk of developing implant loosening during rehab exercises. One proposed method is to apply a screw in the anterosuperior region of the tibial tray to fix the tray to the bone. An alternative method is to include a frontal flange to the tibial tray and screw the flange on the anteromedial aspect of the proximal tibia. To date, little is known about the effectiveness of these two fixation techniques. It is yet to be determined whether these two fixation mechanisms could enhance stability of the PKR during rehab exercises requiring knee flexion.

Finite element analysis (FEA) is ideal to analyze stresses and strains in complex biological structures [20]. The application of this method requires developing a model which represents the real structure in essential features. The model is mathematically divided into little elements connected in nodal points. Computer programs are used to calculate the stresses and strains in the elements and nodes after material properties are assigned and mechanical loads are applied. To date, advanced technologies make personal computers capable of running sophisticate FEA programs and providing results in a matter of hours or days. Thus, it is feasible to investigate the influence of implant design on stress and strain profile at the implant-bone interface via running FEA on a personal computer.

The purpose of this study is two folds. Firstly, to use FEA to determine whether a typical axial mechanical load applied in the posterior region of the PKR results in tension in the anterior implant-bone interface. Secondly, to use FEA to quantify the effects of external fixation mechanisms such as a screw design or a frontal flange design on mechanical stress/strain at the PKR implant-bone interface during weight-bearing knee exercise. It is hypothesized that a posterior axial load would lead to increased tensile loading in the anterior region of the implant-bone interface. It is further hypothesized that external fixation mechanisms could reduce the tensile loading in the anterior region of the implant-bone interface during weight-bearing knee exercise.

## 2. Methods

The left tibia of a healthy male (age = 21 yr., body mass = 78 kg, body height = 181 cm) was CT scanned (GE Light Speed VCT, GE Corp, USA). Slice thickness was 0.625 mm with a 15 cm × 15 cm field of view. The scan parameters were 120 kVp and 140 mAs. Images were reconstructed at 512 × 512 pixels.

CT scans were segmented in MIMICS 14.0 (Materialise, Leuven, Belgium) and a surface mesh was generated. The surface mesh was used as boundaries for the automatic formulation of a solid mesh using hexahedral elements in MARC 2010 (MSC Software, Santa Ana, CA, USA). The mesh consisted of 2 mm × 2 mm × 2 mm elements. Material properties were assigned to the solid tibia. In the longitudinal direction, Young's modulus and the shear elastic modulus of the bone were set at 17 GPa and 10 GPa, respectively. In addition, Young's modulus and the shear modulus of the bone were assumed to be transversely isotropic with values of 5 and 3.5 GPa, respectively [21, 22]. Poisson's ratio was set at 0.3, and bone density was set at 1.9 g/cm^3^.

**Figure 1:**
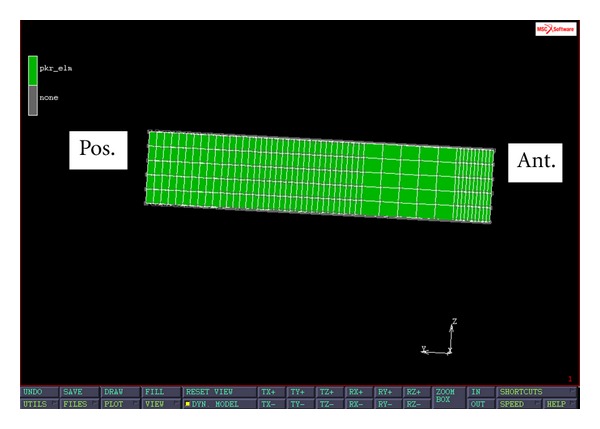
Sagittal view of a PKR model with no external fixations.

**Figure 2:**
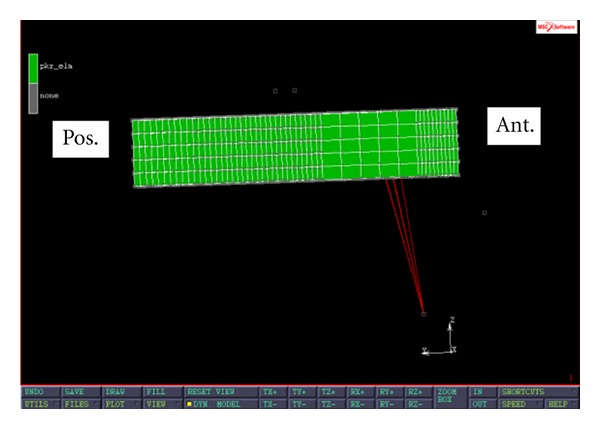
Sagittal view of a modified PKR model with a superior screw design. Red lines in the FE model are RBE2 nodal ties representing a superior screw.

**Figure 3:**
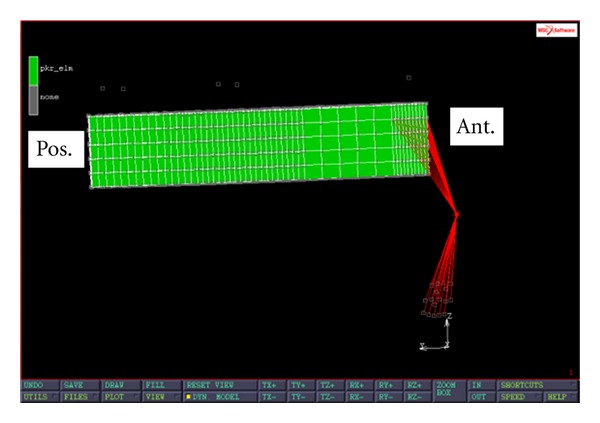
Sagittal view of a modified PKR with a frontal flange design. Red lines in the FE model are RBE2 nodal ties representing a frontal flange.

After the 3D tibia was reconstructed, a typical surgical procedure was simulated in MIMICS (Materialise, Leuven, Belgium) to remove the medial tibial plateau. The thickness of the bone cut was 10 mm. The 3D tibia mesh consisted of 45811 hexahedral elements and 53141 nodes. Three simplified PKR tibial trays were designed. The first model represented a PKR tibial tray with press-fit pegs (Figure 1). The FE PKR model was glued to the tibial plateau to mimic the effect of the press-fit pegs. The second model was a modification of the first model with a superior screw (located 10 mm posteriorly from the front edge of the tray) for stabilizing the tray to the tibial plateau (Figure 2). In this FE PKR model, rigid-body-element nodal ties (RBE2) running from the superior-anterior part of the PKR to the tibial plateau were used to simulate the effect of a superior screw stabilizing the PKR on the bone. The third model was also a modification of the first model with a frontal flange design (Figure 3). One end of the flange was attached to the front edge of the tray. The other end of the flange was screwed to the medioanterior surface of the proximal tibia to externally stabilize the tray. In this FE PKR model, RBE2 nodal ties were used to simulate the effect of the anterior flange screwed on the anteromedial tibia surface.

**Figure 4:**
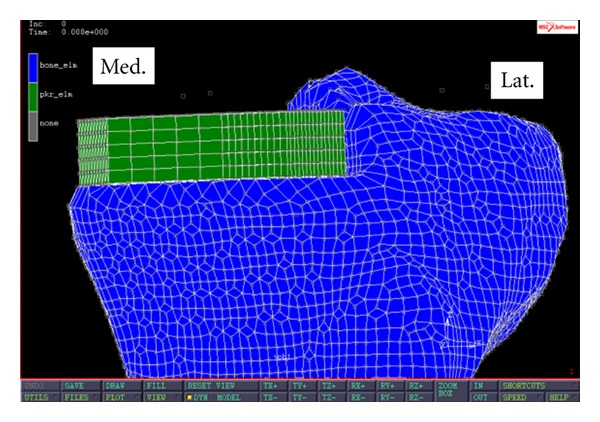
Frontal view of a left tibia with a medial PKR implanted.

The tibial tray of each PKR was solid meshed in MARC 2010 (MSC Software, Santa Ana, CA, USA). The 3D model of the PKR tibial tray consisted of 4750 hexahedral elements and 5826 nodes. Material properties of Cobalt-Chromium (CoCr) were assigned to the tibial component. Young's modulus was set at 195 Gpa; Poisson's ratio was set at 0.3 [23]. Material density was set at 8.85 g/cm^3^. Each PKR model was glued to the medial tibial plateau where bone was removed. The gluing force at the PKR implant and bone interface simulated the effect of the press-fit pegs in a real setting.  Figure 4 shows the frontal view of a PKR tibial tray implanted on the left medial plateau.

Boundary conditions were applied to the tibia and the PKR. The distal end of the tibia was fixed. Medial axial load of 1800 N was applied in the posterior region of the PKR, which was 15 mm posterior to the center of the PKR. Lateral axial load of 1200 N was applied in the posterior aspect of the lateral tibial plateau, which was 15 mm posterior to the center of the lateral plateau. The axial loads chosen were typical loads during stair climbing, squat, or lunge activities [24, 25]. Static FEA was performed to quantify the maximum principal stress/strain at the implant-bone interface when axial loads were applied.

## 3. Results

**Figure 5:**
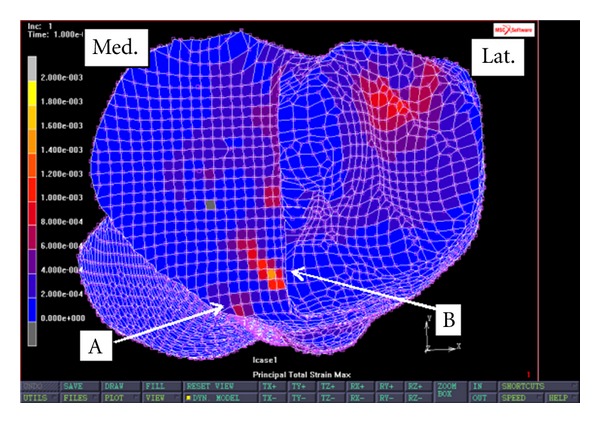
Maximum principal strain at implant-bone interface of a medial PKR knee with no external fixations. “A” indicates the first region with high tensile strain. “B” indicates the second region with high tensile strain.

Figure 5 shows the profile of maximum principal strain at the implant-bone interface during the FEA simulation on the first PKR with no external fixations. Two separate regions with high tensile strain can be identified in the anterior implant-bone interface. The first region is near the anterior edge of the implant-bone interface. The average maximum principal strain in this region is 750 microstrains; the maximum principal strain in this region ranges from 482 microstrains to 1265 microstrains. The second region is located in the anterior aspect of the implant-bone interface and is near the midline of the tibial plateau (Figure 5). The average maximum principal strain in this region is 1198 microstrains; the maximum principal strain in this region ranges from 493 microstrains to 2505 microstrains.

**Figure 6:**
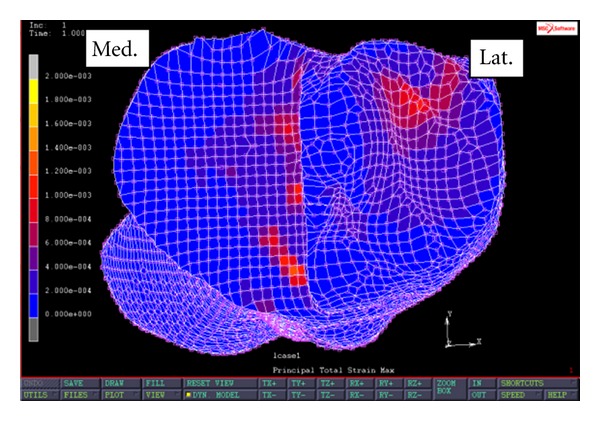
Maximum principal strain at implant-bone interface of a PKR knee with a superior screw design.

Figure 6 shows the profile of maximum principal strain at the implant-bone interface when the FEA simulation was performed on the modified PKR with a superior screw design. The average maximum principal strain in the first region is 511 microstrains. The maximum principal strain in the first region ranges from 308 to 987 microstrains. In addition, the average maximum principal strain in the second region is 851 microstrains. The maximum principal strain in the second region ranges from 201 microstrains to 2305 microstrains. When compared to the PKR model without external fixations, the screwed PKR model exhibits 32% and 29% reductions in maximum principal strain in the first and the second regions, respectively. Specifically, the strain reduction in the first region ranges from 18% to 55%, and the strain reduction in the second region ranges from 4% to 80%.

**Figure 7:**
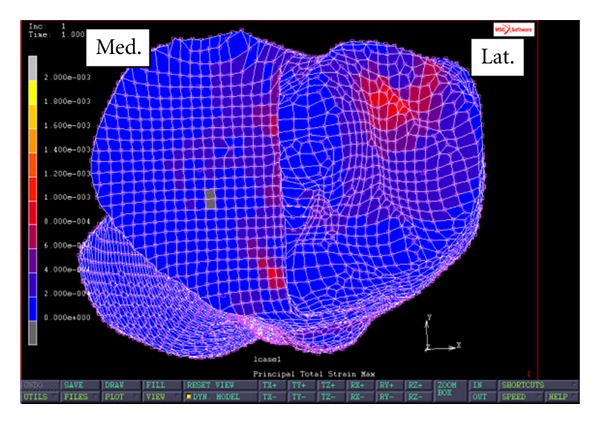
Maximum principal strain at implant-bone interface of a PKR knee with a frontal flange design.

Figure 7 shows the profile of maximum principal strain at the implant-bone interface when the FEA simulation was performed on a modified tibial implant with a frontal flange design. The average maximum principal strain in the first region is 267 microstrains. The maximum principal strain in the first region ranges from 22 to 480 microstrains. Furthermore, the average maximum principal strain in the second region is 633 microstrains. The maximum principal strain in the second region ranges from 313 microstrains to 1133 microstrains. When compared to the PKR design without external fixations, the flanged PKR demonstrates smaller maximum principal strain in the first region (64% less) and in the second region (47% less). Specifically, the strain reduction in the first region ranges from 42% to 95%, and the strain reduction in the second region ranges from 30% to 56%.

## 4. Discussion

In this study, we examined three cementless PKR tibial tray designs. The first design depended on press-fit pegs embedded into the tibial plateau for stability. The second design was a modification of the first design with an addition of a superior screw. The third design was also a modification of the first design with a frontal flange added. An FEA was performed to examine the influence of the PKR design on the stress/strain at the anterior region of the implant-bone interface. The same loading conditions and boundary conditions were applied during each of the FEA simulation. The tensile strains in the anterior region of the PKR implant-bone interface were then examined after the FEA.

We had hypothesized that when axial loads were applied in the posterior region of the first PKR model, there would be increased tensile strain in the anterior region of the implant-bone interface. Indeed, we had identified that there were areas in the anterior region of the implant-bone interface that experienced large tensile strains. The first region showing large tensile strain was near the anterior edge of the implant-bone interface. The second region showing increased tensile strain was found to be posterior to the first region and near the midline of the tibial plateau. Large tensile strains found in the anterior region of the implant-bone interface appear to be a result of the bending effect imposed on the tibia during the simulated weight-bearing exercise.

We also hypothesized that a PKR design with a superior screw would effectively reduce the tensile strain at the anterior implant-bone interface. Our FEA simulation showed that the screw fixation resulted in decreases of tensile strain in the anterior implant-bone interface. In particular, when compared to the PKR model with no external fixations, the screwed PKR showed an average strain reduction of 32% in the first high-strain region and 29% in the second high-strain region. Thus, it appears that the superior screw fixation can alleviate the tensile strain in the anterior region of the implant-bone interface during weight-bearing exercise.

When a frontal flange is added to the tibial tray, a fixation is established between the tibial tray and the medioanterior aspect of the proximal tibia. We had hypothesized that the flanged PKR could effectively reduce the high tensile strain in the anterior region of the implant-bone interface during simulated weight-bearing exercises. This hypothesis was supported. In particular, when compared to the PKR model with no external fixations, the flanged PKR showed an average strain reduction of 64% in the first high-strain region and 47% in the second high-strain region. Thus, it is clear that the frontal flange design can effectively decrease the tensile strain in the anterior region of the implant-bone interface.

In this study, although both the screw design and the flange design can effectively reduce the tensile strain in the high-strain regions, the flange design leads to a greater strain reduction than the screw design. Specifically, the flanged PKR resulted in a 62% strain reduction in the first region compared to a 32% reduction seen in the screwed PKR. Also, the flanged PKR resulted in a 47% strain reduction in the second region compared to a 29% reduction seen in the screwed PKR. Furthermore, the screw design exhibited various effectiveness of strain reduction in the second region. The percentage reduction of tensile strain ranged from 4% to 80%. It is possible that stress concentration at the interface of the screw and bone may contribute to the small effect of strain reduction in some locations within the second region. Interestingly, the flanged PKR demonstrated a uniform effect of tensile strain reduction in the second region. The percentage reduction of the tensile strain was found to be ranged from 30%–56%.

Standard knee rehab programs for PKR patients include weight-bearing exercises such as squatting and lunging. Depending on the type of exercises, the axial load can be as high as 5–7 times of the body weight [26]. The medial compartment of the knee normally experiences higher loading than the lateral compartment [4, 5, 24]. It was found that during squat exercise, the axial loading in medial compartment is 1.5 times of the lateral compartment or 60% of the total axial load in the knee joint [27]. In addition, during knee rehab exercises requiring knee flexion, the axial load is applied in the posterior region of the tibial tray [18, 19]. Thus, for patients with medial PKR, rehab exercises could result in high tensile strain in the anterior implant-bone interface. In this study, the press-fit PKR knee experiences a peak tensile strain of 2500 microstrain in the anterior region. The magnitude of the strain has exceeded the physiological tensile strain in human bone, which is below 1500–2000 microstrain [28–30]. As bone can develop fatigue fracture with relatively few loading cycles when cyclic strains are large, it is possible that the cyclic loading introduced during knee rehab exercises may increase risk of bone microfracture at the implant-bone interface. Thus, it is advisable for patients to avoid rehab exercises resulting in mechanical loading in the posterior region of the PKR knee. In addition, even after new bone tissue has fully grown into the implant surface, it is cautious for patients to regularly engage in exercises such as squatting and lunging, which would lead to large mechanical loads in the posterior region of the PKR. Not surprisingly, the tensile strains at the implant-bone interface of the screwed PKR knee are mainly within the range of the physiological bone strain with an exception of one small area showing a relatively high strain of 2300 microstrains. This reflects that the effect of a superior screw on lowering bone strain varies from region to region. It is possible that the superior screw may not be effective in reducing strain in some areas of the implant-bone interface. Thus, for the longevity of a PKR knee, patients with a screwed PKR may consider avoiding exercises requiring knee flexion with large posterior knee loading. In this study, we found that the flanged PKR could sustain typical posterior knee loading without having large strains. Therefore, patients with a flanged PKR can safely engage in knee rehab exercises involving lunge and squats, which could help the surgical knee to regain function quickly. In the long run, with a flanged PKR, patients can regularly perform physical activities involving knee flexion and habitual posterior knee loading (e.g., squatting and lunging) without risk of bone microdamage at the implant-bone interface.

Knee OA is often limited in medial knee compartment and patellofemoral joint [3]. Cementless PKR has been widely used to treat medial knee OA and patellofemoral OA. Compared to TKR, PKR requires less bone cut and a shorter hospital stay. With a PKR, patients can retain cruciate ligaments and normal joint geometry to achieve satisfied knee mechanics [8, 12–15]. Most importantly, patients can achieve fast recovery by engaging in aggressive rehab exercises to regain knee strength and good knee mechanics. However, in the first two to three months postsurgery, bone tissue is in the process of remodeling and new bone tissue is growing into the implant surface. During bone remodeling, the strength of the bone tissue at the implant-bone interface is weakened. Thus, large mechanical load may result in bone microdamage and implant loosening. In this study, we determine that a typical axial load during knee exercises can lead to bending effect on the anterior aspect of the tibia and result in increased tensile strain in the anterior region of the implant-bone interface. If the implant-bone interface experiences repetitively large tensile strain during rehab exercise, implant loosening is likely to occur due to microdamage of weakened bone tissue at the implant-bone interface. Thus, it is important to introduce external fixation mechanisms to stabilize the implant during knee rehab exercises. In this study, we confirmed that external fixation mechanisms enhance stability of the cementless PKR during simulated knee rehab exercises. Both the superior screw design and the frontal flange design have demonstrated effectiveness of reducing tensile strain in the anterior region of the implant-bone interface. In particular, the flanged PKR showed lowered tensile strain within physiological strain range. Therefore, to better alleviate tensile strain at the implant-bone interface, external fixation mechanisms such as a superior screw or a frontal flange should be incorporated into future cementless PKR designs.

There are a couple of limitations associated with this study. Firstly, a phantom was not used during the CT scan. Without phantom data, it was difficult to establish an accurate relationship between bone density and material properties for both the cortical and cancellous bone. Although it would be ideal to model the tibia plateau with separate cancellous bone properties, it was acceptable to model the entire tibia with linear elastic and transversely isotropic material [21, 22]. Because the mechanical behavior of cancellous bone was found to be similar to that of cortical bone, FEA studies have been conducted to analyze fatigue life of cancellous bone with cortical bone's properties applied [31]. Results from those studies are in reasonable agreement with experimental testing [31]. Furthermore, as comparisons were made among the three PKR designs based on the same tibial model, the trend of mechanical differences exhibited among the three designs is valid. Secondly, a subject-specific tibial model was used in this study. The strain values from the FEA simulation can only be applied to the tibia bone chosen. As the geometry of the tibia bone may vary from person to person, it is possible that for a given mechanical load, the strain level at the implant-bone interface may be different from person to person. However, this does highlight the importance of using subject-specific FE models for evaluating the mechanics of individual PKR knees.

In summary, posterior axial loading could lead to increased tensile strain in the anterior interface between the PKR and tibia bone. Both the screwed and flanged PKRs can reduce the tensile strain in the anterior region of the implant-bone interface. Flange design demonstrates a more uniform effect of reducing tensile strain in the anterior region of the implant-bone interface than the screw design.
